# Exploring the Differential Impact of Melatonin Timing Administration (Day vs. Night) on Mitigating Cerebellar Damage Caused by Monosodium Glutamate in Rats

**DOI:** 10.1002/jbt.71108

**Published:** 2026-09-14

**Authors:** Alaa Gamal Ahmad, Noha M. Abd El‐Fadeal, Horeya Erfan Korayem Arafat, Dalia Ibrahim Ahmed, Rasha Atta

**Affiliations:** ^1^ Department of Human Physiology Faculty of Medicine, Suez Canal University Ismailia Egypt; ^2^ Department of Medical Biochemistry and Molecular Biology Faculty of Medicine, Suez Canal University Ismailia Egypt; ^3^ Department of Biochemistry Ibn Sina National College for Medical Studies Jeddah Saudi Arabia; ^4^ Oncology Diagnostic Unit, Faculty of Medicine, Suez Canal University Ismailia Egypt; ^5^ Department of Histology and Cell Biology Faculty of Medicine, Suez Canal University Ismailia Egypt; ^6^ Center of Excellence in Molecular and Cellular Medicine (CEMCM), Faculty of Medicine, Suez Canal University Ismailia Egypt

**Keywords:** cerebellar ataxia, chronotherapy, melatonin, monosodium glutamate, NRF2/HO‐1 pathway

## Abstract

In tune with the light/dark cycle of the environment, melatonin is secreted on a circadian rhythm. After the lights go out, circulating melatonin levels gradually rise and peak in the middle of the dark phase. Herein, we studied the possible protective timing of melatonin administration on monosodium glutamate‐induced cerebellar ataxia in rat models. Thirty‐two adult albino male were randomly categorized into four groups: control group, MSG group (4 g/kg/day), day‐time melatonin group (MSG + melatonin, 10 mg/kg/day at 9.00 a.m.), and night‐time melatonin group (MSG + melatonin, 10 mg/kg/day at 9.00 p.m.). After 10 days of injections, behavioral assessments and serum melatonin were measured. Then rats were sacrificed, cerebellar immunohistopathology, oxidative stress (GPX, GR, NO, iNOS), gene expression of pro‐inflammatory genes (PI3K, TNF‐α, IL‐6) and antioxidant genes (NRF2/HO‐1) were assessed. Administration of MSG significantly caused motor deficits, increased levels of NO, INOS, PI3K, TNF‐α, and IL‐6 levels (*p* < 0.05), while significantly decreased GPX, GR, NRF2/HO‐1, and serum melatonin level (*p* < 0.05). Histological analysis revealed that MSG exerted degenerative effects, including the presence of pyknotic Purkinje cells, with strong positive reactions for COX2. However, melatonin administration improved these parameters in both treated groups with the superiority of the night melatonin in improving motor behaviors, histological parameters, decreasing COX2 levels, INOS, PI3K, and increasing the expression of NRF2 levels, and increasing the serum melatonin levels. Night‐time melatonin administration was more effective in protection against motor incoordination and cerebellar damage than day‐melatonin administration.

## 1 Background

Cerebellar ataxia encompasses a variety of inherited and acquired neurodegenerative disorders [1]. Neurodegenerative disorders are a significant social and medical problem nowadays affecting individuals across all age groups. The burden of society's inability to treat neurodegenerative diseases effectively is enormous [2]. Cerebellar Ataxia patients usually have a poor quality of life with several pathophysiological manifestations such as coordination disorders, oculomotor deficits, dysmetria, and kinetic tremors. There isn't currently effective drug that can completely halt or prevent neuronal degeneration or improve cerebellar function [3].

The salt form of l‐glutamic acid, monosodium glutamate (MSG), which is commonly utilized as a flavor enhancer worldwide, has been proposed as the cause of the neuronal degeneration known as “glutamate excitotoxicity.” Nonetheless, it has been argued that over 40 years of research on humans has consistently failed to uncover indisputable proof of MSG harm to organs [4]. Several lines of research on animal models appeared to show the deleterious effect of MSG on many organs, including kidney, liver, thymus, ovaries, and cerebellum [5].

Melatonin is an amphiphilic indoleamine (*N*‐acetyl‐5‐methoxytryptamine), is an endogenous tryptophan‐derived neurohormone, and is well known for being an effective free radical scavenger that is largely produced and released by animals’ pineal glands during the dark phase of the light‐dark cycle [6]. Melatonin can be released at number of CNS locations outside the pineal gland, such as cerebral cortex, hypothalamus, nucleus gracilis, cerebellum, medulla oblongata, and retina, modulating number of neurophysiological functions [7].

Additionally, earlier research suggested that melatonin may function as a neuroprotective chemical in both acute brain injuries such as cerebral ischemia and long‐term neurodegenerative diseases [8]. In addition, in both in vivo and in vitro experimental models of lipopolysaccharide (LPS)‐induced inflammation, melatonin inhibited nuclear factor kappa B (NF‐κB) translocation and matrix metallopeptidase‐9 (MMP‐9) activation [9].

Superoxide dismutase, catalase, and glutathione peroxidase are just a few of the antioxidant enzymes that this indoleamine increases the activity and its expression in order to reduce oxidative stress [10]. Melatonin secretion, however, has a significant circadian pattern, which is characterized by a greater secretion at night and decreased or absent secretion throughout the day [11]. Some studies have been done to discover different therapeutic effect of melatonin depending on timing dose whether at day or night at other diseases rather than neurodegenerative diseases like ataxia [12].

Up to our knowledge, the protective potentials of melatonin depending on its administration timing during day/light cycle in MSG‐induced cerebellar ataxia remains unexplored. So, this research compared the different protective effects of melatonin induced cerebellar ataxia by MSG at day and night times.

## 2 Methods

### 2.1 Chemical and Drug Preparation

Monosodium Glutamate (Monosodium l‐glutamate monohydrate, Sigma Aldrich, USA) was prepared daily by dissolving 30 g of MSG in 50 mL saline (0.9% NaCl), Melatonin (Sigma Aldrich, USA) was prepared by dissolving 1 g of melatonin in 1 mL (100%) ethanol then diluted with 99 mL saline (0.9% NaCl).

### 2.2 **Experimental Design: (**Figure 1 **)**

A total of 32 adult albino rats weighing (150–200 g) were included in the study. The rats were housed in a normal light‐dark cycle in a hygienic environmental condition. They were kept for 1 week acclimatization before the start of the experiment.

**Figure 1:**
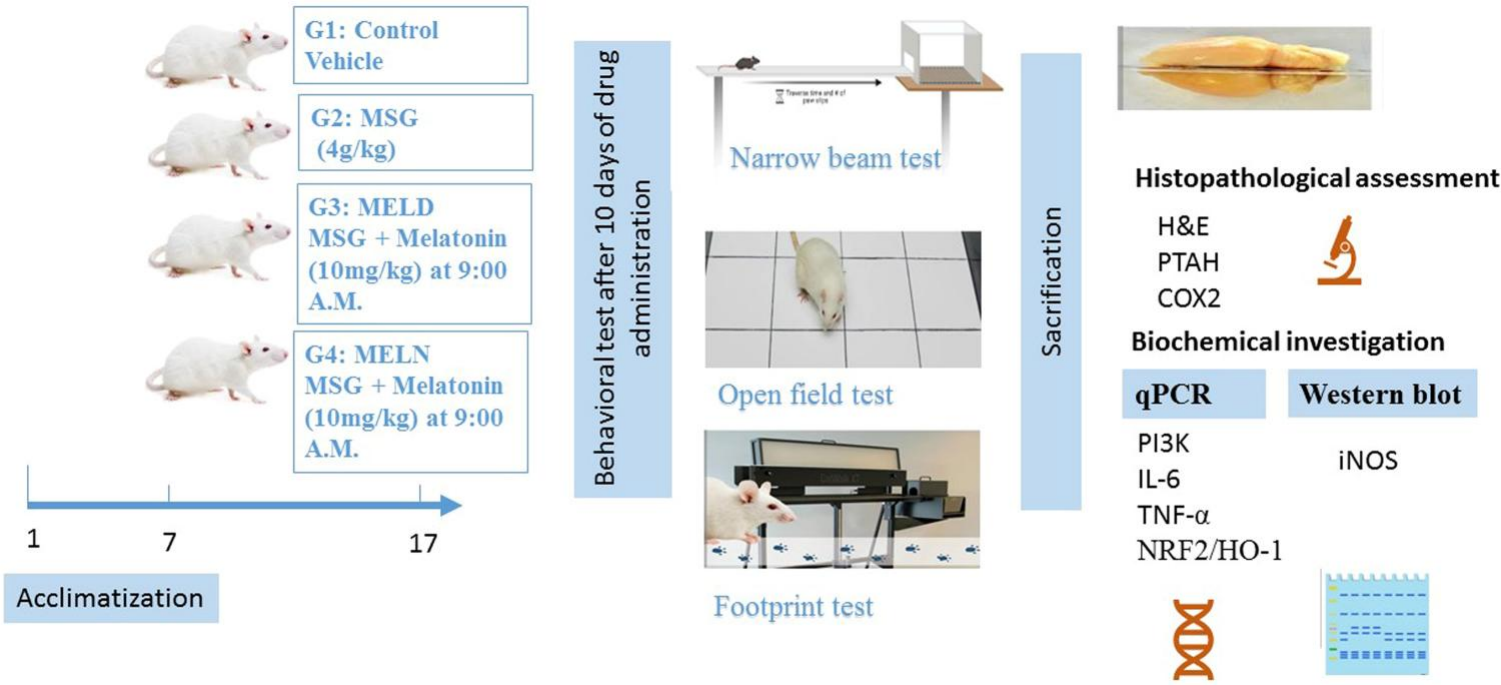
Schematic diagram representing the study design. This figure was created by PowerPoint program.

### 2.3 Study Groups

Rats were randomly assigned to 4 groups with 8 rats in each group: *Control group*, rats received 2 mL/kg of 1% ethanol (melatonin vehicle) by intraperitoneal injection daily for 10 days. *MSG group*, rats were orally administrated MSG 4 g/kg body weight by intragastric gavage daily for 10 days [13]. *Day‐time melatonin group (MELD)*, rats were administrated the same previous dose of MSG after intraperitoneal injection of melatonin 10 mg/kg body weight [14, 15] for 10 days [16] at 9:00 a.m. [12]. *Night‐time melatonin group (MELN)*, rats were administrated the same previous dose of MSG after intraperitoneal injection of melatonin 10 mg/kg body weight for 10 days at 9:00 p.m. [12].

### 2.4 Neurobehavioral Assessment of the Motor Function

After 10 days of treatment by MSG and Melatonin, motor assessment of ataxia was done.

#### 2.4.1 Narrow Beam Test

Rats were trained to traverse a narrow wooden beam (100 cm long, and 1 cm wide) to reach the opposite platform. The time was recorded when all 4 ft. were fully planted on the finishing platform at the other end of the beam, with a maximum limit of 2 min. The start line must be crossed within 1 min from release, or else the test was aborted, and the maximum time was recorded for that trial. Three attempts comprised a testing session. The time taken to cross the beam and the number of hind limb slippages were recorded [17].

#### 2.4.2 Open Field Test

Rats were introduced individually in an open field arena (113 × 113 × 44 cm) constructed from white painted plywood with a floor marked with black lines forming a 5 × 5 cm pattern. Behavioral parameters were recorded on video for later analysis during 5‐min sessions. The box was cleaned with a 70% ethanol between each trail. Ambulation (the number of squares crossed) and total movement time were recorded as parameters of motor activity [18].

#### 2.4.3 Footprint Walking Tract Test

The apparatus consisted of an open field (60 × 60 × 40 cm) illuminated by a light source in one end, featuring a runway (4.5 cm wide, 42 cm long, borders 12 cm) was arranged to lead out into a dark wooden box (20 × 17 × 10 cm). The rat's forelimbs and hind limbs were dipped in black and red non‐toxic dyes, respectively, to record the footprints. Rats were allowed to traverse a white sheet toward a custom‐built dark escape box, leaving a trace of their paw prints on the sheet. Six middle steps of a series of steps were analyzed for forelimb stride length, hind‐limb stride length (HSL), forelimb base width, hindlimb base width and overlap [19, 20, 21].

### 2.5 Cerebellar Preservation

At the end of the experiment, Animals were first deeply anesthetized with ketamine (80 mg/kg) and xylazine (10 mg/kg), followed by blood sampling then transcardial perfusion with saline followed by 4% paraformaldehyde and finally euthanasia was completed by decapitation before tissue collection. The cerebellum of each animal was divided into two halves. The first halves were fixed in a 10% formalin solution for 24 h for subsequent immunohistopathological processing. The other halves were preserved at –80°C for oxidative stress, PCR, and Western blot analysis.

### 2.6 Histological Processing, Staining, and Morphometric Analysis

Cerebellar tissues were fixed in 10% neutral‐buffered formalin for 24 h. The tissues were dehydrated through a graded ethanol series, cleared in xylene, and embedded in paraffin. The paraffin blocks were processed for light microscopic study. Serial sagittal sections (5–7 µm) were cut using a rotary microtome (Leica RM2235), mounted on poly‐l‐lysine‐coated glass slides, and stained with hematoxylin and eosin (H&E) for general histoarchitecture, Phosphotungestic acid hematoxylin (PTAH) for dendrites, and Cresyl fast violet (Nissl stain) for visualization of Nissl granules in Purkinje cell perikarya. All stained sections were examined by an observer blinded to the sample labels [22].

Quantitative histopathological assessment of Purkinje cells in the Purkinje cell layer included evaluation of normal cells, shrunken cells, cytoplasmic changes (darkly stained or vacuolated cytoplasm), and loss of cellular continuity.

For immunohistochemistry, sections were deparaffinized, underwent heat‐induced antigen retrieval in citrate buffer (pH 6.0), and endogenous peroxidase activity was blocked with 0.006% H_2_O_2_. Sections were incubated overnight at 4°C with a rabbit polyclonal anti‐cyclooxygenase‐2 (COX2) primary antibody (Sigma‐Aldrich), followed by incubation with a biotinylated secondary antibody and streptavidin‐HRP complex (Novacastra). Diaminobenzidine (DAB) was used as the chromogen, and sections were counterstained with Mayer's hematoxylin.

Quantitative morphometric analysis was performed on digitized images captured under consistent conditions (400× magnification) using ImageJ software (NIH, USA). The following parameters were measured:

– Dendritic length of Purkinje cells extending up into the molecular layer (from PTAH‐stained sections).
– The mean area percentage of COX2 immunostaining in the cerebellar cortex, determined by color‐threshold segmentation of DAB‐positive regions.

For each animal, three non‐overlapping fields from three separate sections were analyzed, and the mean value was used for statistical comparison.

### 2.7 Biochemical and Molecular Assays

#### 2.7.1 Quantitative Real‐Time PCR (qRT‐PCR)

Total RNA was isolated from the samples using the RNeasy Mini Kit (Qiagen, Cat. No. 74134). RNA purity was confirmed with a NanoDrop 2000c spectrophotometer, yielding A260/A280 ratios between 1.8 and 2.0. Subsequently, cDNA was generated from the purified RNA via reverse transcription employing the QuantiTect Reverse Transcription Kit (Qiagen, Cat. No. 205311).

Quantitative reverse transcription PCR (qRT‐PCR) was carried out in triplicate reactions with the QuantiTect SYBR Green PCR Master Mix (Qiagen, Cat. No. 204141) on a StepOnePlus Real‐Time PCR System (Applied Biosystems). The thermal cycling protocol consisted of an initial step at 95°C for 15 min, followed by 45 cycles of denaturation at 95°C for 15 s, annealing at 60°C for 30 s, and extension at 72°C for 40 s. Primer sequences used are provided in Table 1. Target gene expression levels were normalized to the endogenous control β‐actin and quantified using the 2^−ΔΔCt^ method [23].

**Table 1:** Primer sequences used for qRT‐PCR.

Genes	Forward Primer: (5′ → 3′)	Reverse Primer: (5′ → 3′)
PI3K	ACCATCAGTGGCTCTGCGGTTT	GTGGTCTTCTGGGAACTCACCT
TNF‐α	GGTGCCTATGTCTCAGCCTCTT	GCCATAGAACTGATGAGAGGGAG
IL‐6	TACCACTTCACAAGTCGGAGGC	CTGCAAGTGCATCATCGTTGTTC
HO‐1	CCAGGCAGAGAATGCTGAGTTC	AAGACTGGGCTCTCCTTGTTGC
NRF2	CAGCATAGAGCAGGACATGGAG	GAACAGCCGTAGTATCAGCCAG
β‐actin	CATTGCTGACAGGATGCAGAAGG	TGCTGGAAGGTGGACAGTGAGG

#### 2.7.2 Western Blot Analysis for iNOS

Cerebellar samples were lysed in a cold RIPA buffer (50 mM Tris‐HCl pH 8.0, 150 mM NaCl, 1% NP‐40, 0.1% SDS, 1 mM sodium orthovanadate, 1 mM NaF) containing a commercial protease inhibitor cocktail. The lysates were rotated at 4°C for 30 min and then centrifuged at 16,000 × *g* for 20 min at 4°C to pellet debris. The protein concentration of the supernatant was measured with a bicinchoninic acid (BCA) assay. For immunoblotting, equal protein aliquots (25–50 µg) were combined with 2× Laemmli buffer, boiled at 95°C, and separated by electrophoresis on 10% SDS‐polyacrylamide gels. A prestained protein ladder was included as a molecular weight standard. After separation, proteins were transferred onto PVDF membranes using a wet transfer system at 100 V for 1–2 h. Membranes were blocked with 5% non‐fat milk in TBST and subsequently probed overnight at 4°C with primary antibodies against iNOS and β‐actin diluted in a 5% BSA solution. Following washes, membranes were incubated for 1 h at room temperature with appropriate HRP‐conjugated secondary antibodies. Signal detection was performed with an enhanced chemiluminescence (ECL) substrate, and images were captured digitally. Band intensities were quantified by densitometry, with iNOS levels normalized to the β‐actin signal.

#### 2.7.3 Serum Melatonin Measurement

Serum melatonin levels were measured with a competitive ELISA kit designed for rat samples (Elabscience, Catalog No. E‐EL‐R0031), following the provided protocol. In brief, 50 µL of serum or standard was added in duplicate to pre‐coated wells, followed by 50 µL of biotinylated detection antibody. After a 45‐min incubation at 37°C and washing, 100 µL of HRP‐conjugated avidin was added and incubated for 30 min at 37°C. Following another wash, 90 µL of TMB substrate was added, and the plate was incubated in the dark at 37°C for 15 min. The reaction was stopped with 50 µL of stop solution, and the absorbance was measured at 450 nm with a 630‐nm reference using a microplate reader. Concentrations were determined by interpolation from an 8‐point standard curve, with all samples analyzed in duplicate.

### 2.8 Statistical Analysis

All quantitative results are reported as the mean ± standard error of the mean (SEM). Data distribution normality was evaluated with the Shapiro Wilk test, and variance homogeneity was confirmed using Levene's test. For comparisons across the four experimental groups, a One‐way analysis of variance (ANOVA) was employed. When a significant main effect was found, Bonferroni's post‐hoc test was applied for multiple comparisons. Statistical significance was defined as a two‐tailed *p*‐value < 0.05. Analyses were performed using IBM SPSS Statistics (version 26), and figures were created with GraphPad Prism (version 8.0).

## 3 Result

### 3.1 Melatonin Ameliorates Motor Behavior and Coordination

**Figure 2:**
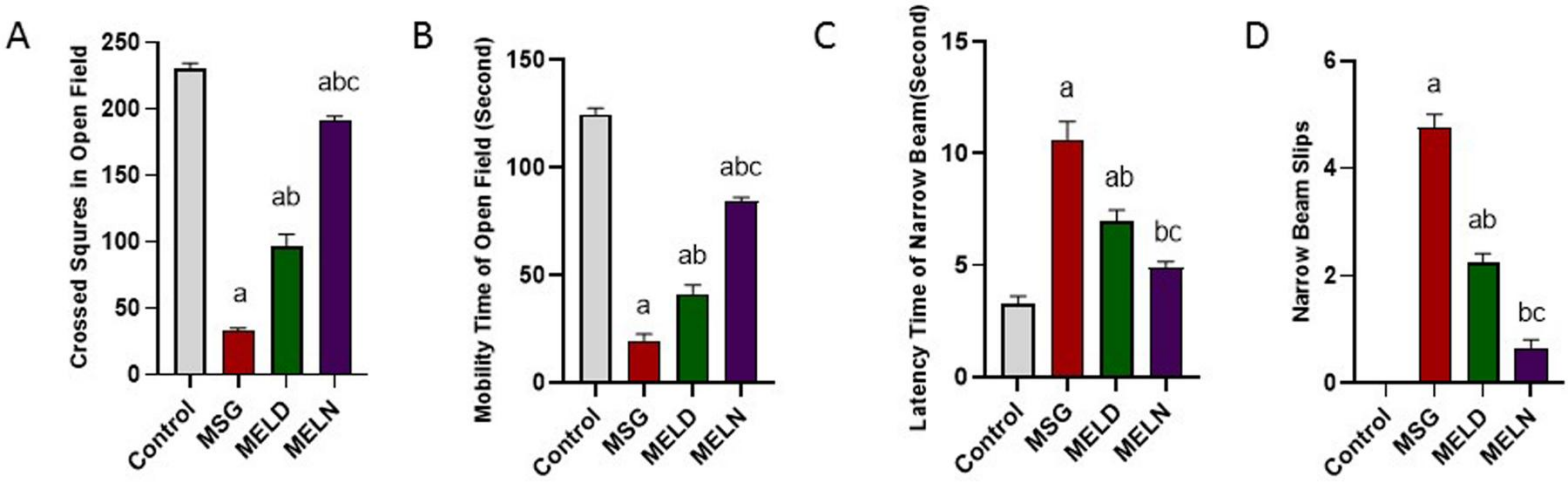
The effect of MSG and melatonin treatment on motor behavior in the experimental groups (*n* = 8/group). (A) Number of crossed squares in open field test, (B) Mobility time on open field test, (C) Latency time on narrow beam test, and (D) Narrow beam slips. Control: Control group, MSG: Monosodium glutamate group, MELD: Day‐time melatonin group, MELN: Night‐time melatonin group. All data presented as Mean ± SEM. All data were analyzed using One‐way ANOVA followed by Bonferroni post‐hoc test. a: statistically significant compared to control, b: statistically significant compared to MSG group, c: statistically significant compared to Day‐time melatonin group.

Assessment of motor behavior in open field test demonstrated a significant increase in the crossed squares both treated melatonin groups when compared to the MSG group (*p* < 0.05), with a more significant increase in the night‐time melatonin group (*p* < 0.05). In addition, total movement time showed a significant increase in both treated melatonin groups compared to the MSG group (*p* < 0.05), with a more significant improvement in the night‐time melatonin group (*p* < 0.05) (Figure 2A,B).

Narrow beam revealed a significant decrease in the latency time of the day‐time melatonin group compared to the MSG group (*p* < 0.05). The night‐time melatonin group demonstrated a near normal latency time with more significant decrease compared to the MSG and day‐time melatonin groups. Regarding the number of slips, both treated melatonin groups showed a significant decrease compared to the MSG group with more significant improvement in the night‐time melatonin group (*p* < 0.05) (Figure 2C,D).

**Figure 3:**
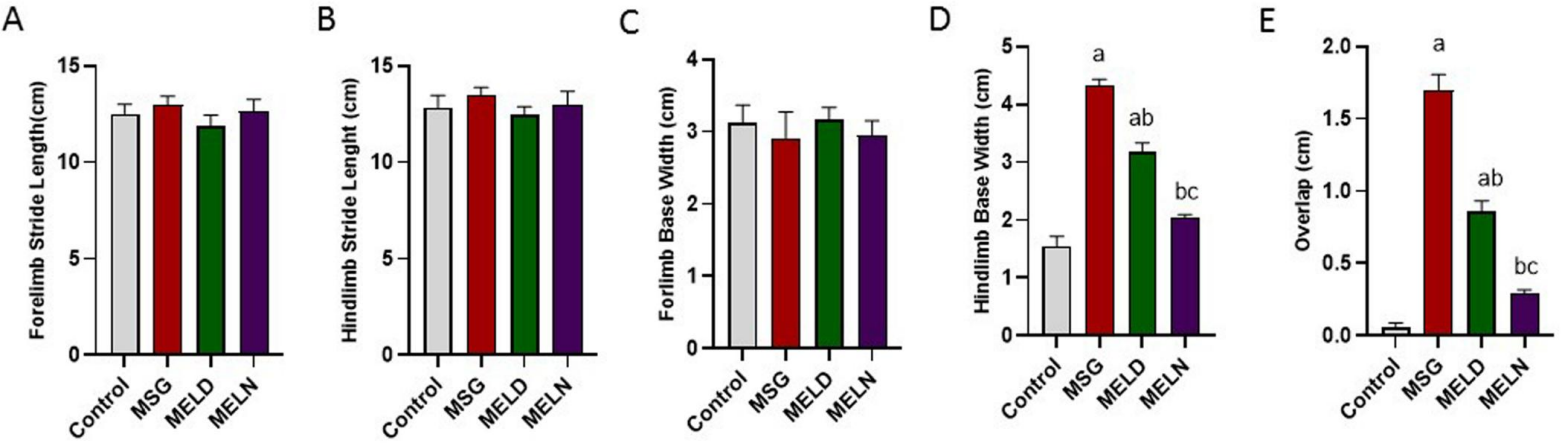
The effect of MSG and melatonin treatment on walking tract footprint in the experimental groups (*n* = 8/group). Control: Control group, MSG: Monosodium glutamate group, MELD: Day‐time melatonin group, MELN: Night‐time melatonin group. All data presented as Mean ± SEM. All data were analyzed using One‐way ANOVA followed by Bonferroni post‐hoc test. a: statistically significant compared to control, b: statistically significant compared to MSG group, c: statistically significant compared to Day‐time melatonin group.

Walking tract footprint analysis showed no significance difference in both limbs stride lengths and the forelimb base width in the four experimental groups (*p* < 0.05) (Figure 3A–C). Regarding the hindlimb base, there was a significant decrease in width in the day‐time melatonin group compared to the MSG group. The night‐time melatonin group demonstrated normalization of the hindlimb base‐width (no significant difference with the control group), with a more significant decrease compared to the day‐time melatonin group (*p* < 0.05) (Figure 3D).

According to the overlap, the day‐time melatonin group showed a significant decrease compared to the MSG group whereas, the night‐time melatonin group showed results similar to the control group (*p* < 0.05) (Figure 3E).

### 3.2 Melatonin Ameliorates Histological Results

#### 3.2.1 H&E Stain in the Cerebellar Tissue

**Figure 4:**
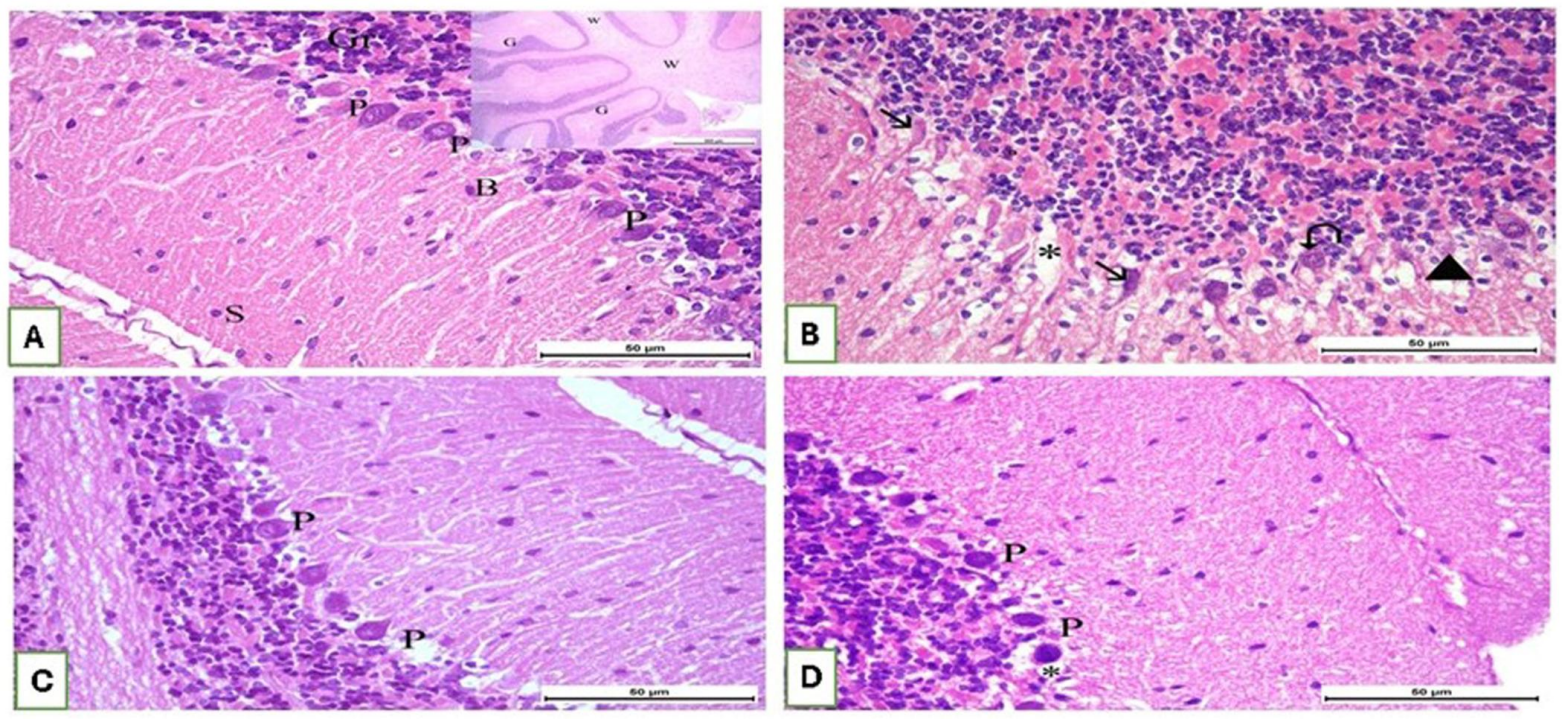
Photomicrograph of sections with H&E stain in the cerebellum. (A) Control group showing the folia, which composed of gray matter (G) and a core of white matter (W) [H&E x100]. A higher magnification showing cerebellar cortex which is showing scattered small stellate (Sc) and basket cells (B) in the molecular layer, the Purkinje cell layer (P); large pyriform cells with vesicular nuclei and the granular layer (Gr [H&E x400]. (B) MSG group showing a normal Purkinje cell (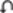), widely displaced, distorted and shrunken Purkinje cell (arrow), leaving vacuoles (*) in intercellular spaces due to complete absence of Purkinje cells. One Purkinje cell has lightly stained cytoplasm and karyolitic nucleus (▶) [H&E x400]. (C) Day‐time melatonin group shows restoration of the normal architecture of the cortex and increased numbers of normal Purkinje cells (P) like that of the control group [H&E x400]. (D) Night‐time melatonin group shows increased number of Purkinje cell (P) and restoration of the normal architectural of the cortex, like that of the control group, but less than group III. Some Purkinje cells are displaced, distorted and shrunken, leaving vacuoles (*) in intercellular spaces [H&E x400].

**Figure 5:**
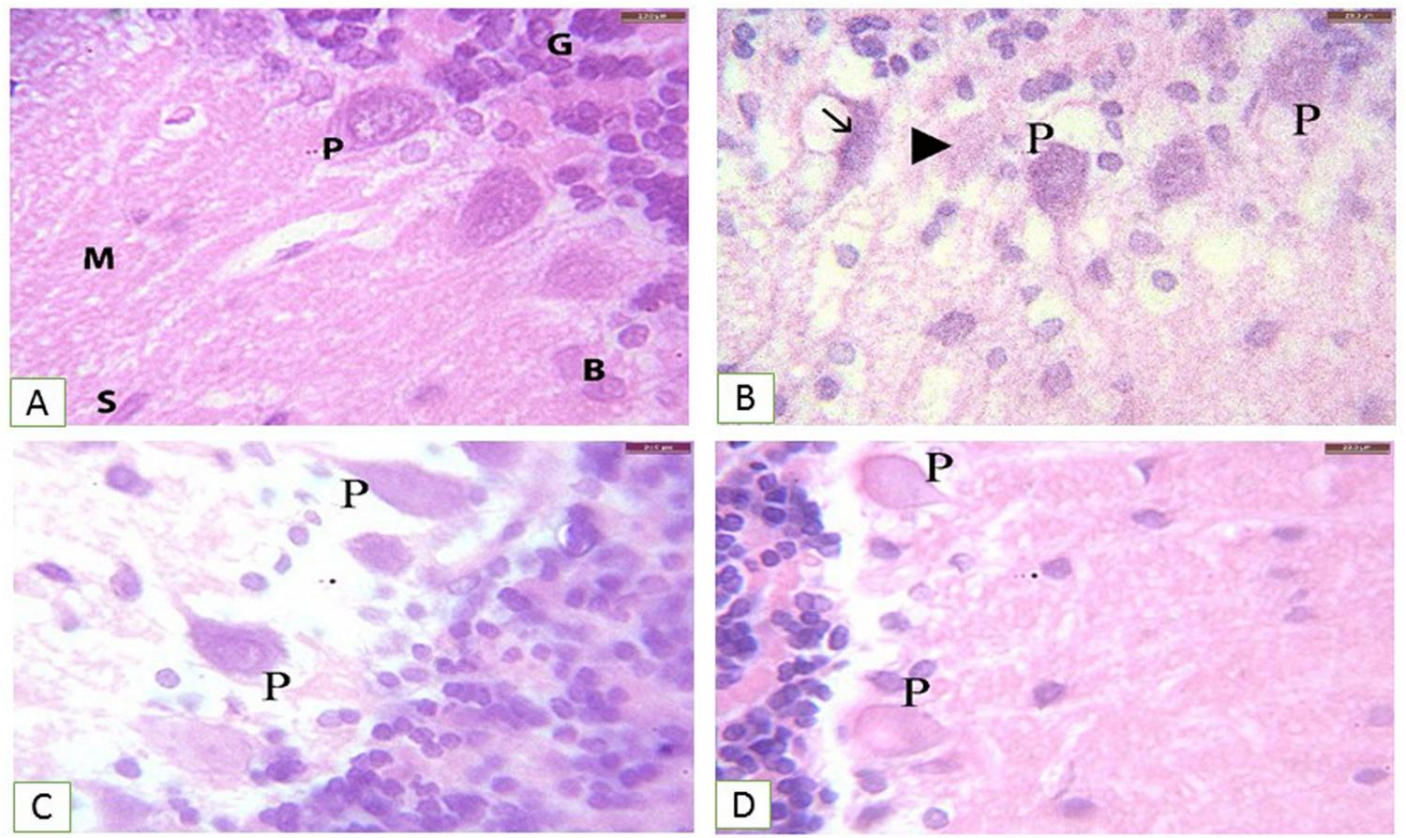
Photomicrograph of sections with H&E stain in the cerebellum with high magnification [H&E x1000]. (A) A higher magnification showing the control group of the cerebellar cortex, which is formed of stellate (S) and basket cells (B) in the molecular layer (M); the Purkinje cell layer (P), which is formed of Purkinje cells, large pyriform cells with vesicular nuclei; and the granular cell layer (G), which is formed of granule cells. B: MSG group showing normal Purkinje cell (P), widely displaced shrunken distorted Purkinje cell (↘), and lightly stained cytoplasm with karyolytic nucleus (▶). (C) Day time melatonin group shows restoration of the normal architecture of the cortex and increased number of the Purkinje cells and restoration of its normal shape. (D) Night time melatonin group shows restoration of the normal architecture of the cortex and increased number of the Purkinje cells and restoration of its normal shape, more than day group.

Hematoxylin and eosin–stained cerebellar sections of the control group (Figures 4A and 5A) revealed rat cerebellar cortex with its three layers: molecular, Purkinje, and granular layers. The molecular layer contained basket cells and smaller stellate cells. The control group showed a relatively uniform structure with a single row of Purkinje neurons between the molecular and granule layers, showing the folia, which is composed of gray matter and a core of white matter.

**Figure 6:**
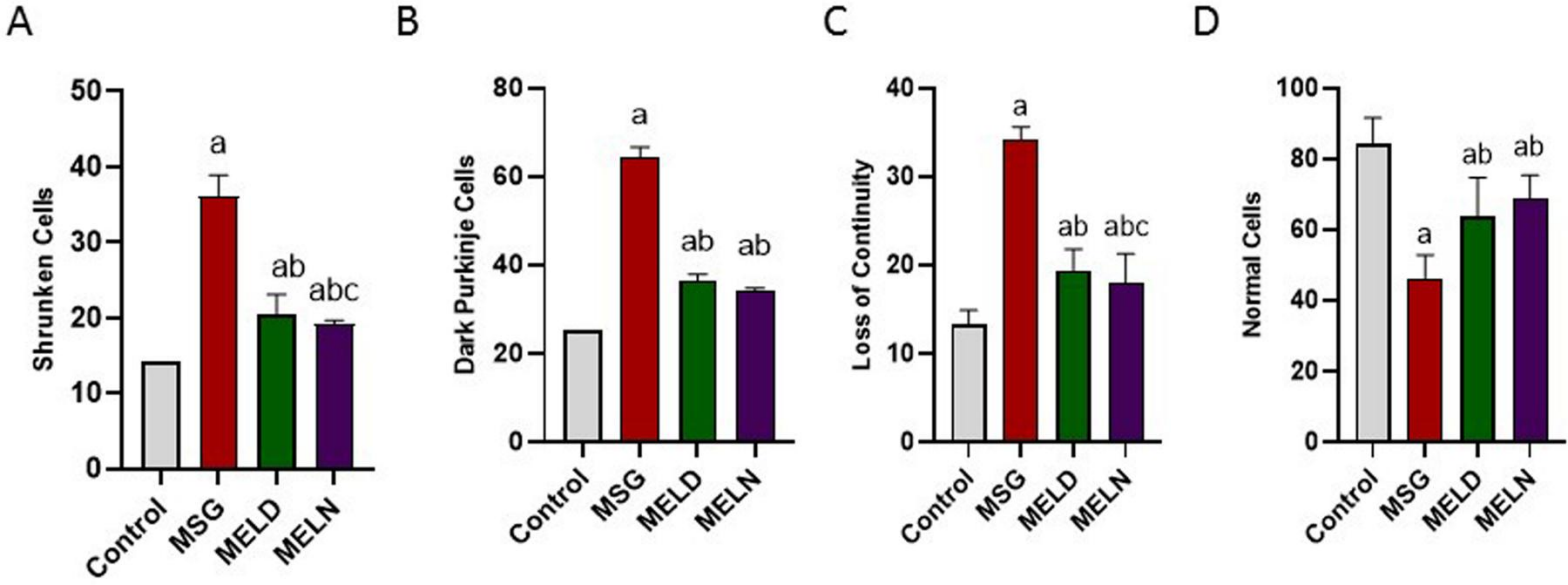
The frequency distribution of the histopathological changes in Purkinje cell layer in the different experimental groups. Control: Control group, MSG: Monosodium glutamate group, MELD: Day‐time melatonin group, MELN: Night‐time melatonin group. All data presented as Mean ± SEM. All data were analyzed using One‐way ANOVA followed by Bonferroni post‐hoc test. a: statistically significant compared to control, b: statistically significant compared to MSG group, c: statistically significant compared to Day‐time melatonin group.

However, in the MSG treated rats (Figures 4B and 5B) the alignment of the Purkinje cells was widely displaced, distorted, showing irregular, scattered somas, shrunken Purkinje cell and leaving vacuoles in intercellular spaces due to complete absence of Purkinje cells. Some of which appeared to be hyperchromatic and shrunken, suggesting pyknosis by H&E stain. The cerebellum of the rats treated with melatonin by day shows an increased number of Purkinje cells and restoration of the normal architectural of the cortex, like that of the control group, but less than the night‐time melatonin group as shown in (Figures 4, 5, and 6).

#### 3.2.2 PTAH Result

**Figure 7:**
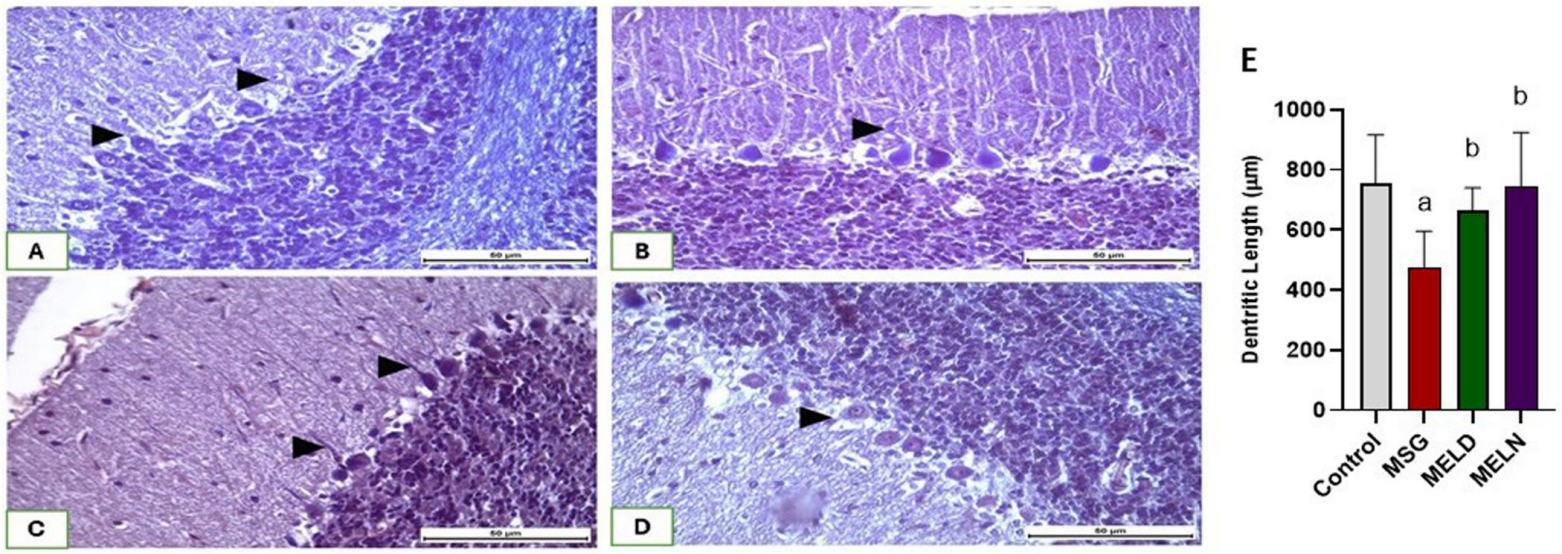
A photomicrograph of sections with the PTAH stain in the cerebellar cortex [PTAH stain X400]. (A) Control group showing delicate dendritic arborizations of Purkinje cells extending up into molecular layer (arrowhead). (B) MSG group showing decreased in length of Purkinje cell dendrites. (C) Day‐time melatonin group showing dendrites of Purkinje cell dendrites, nearly like that of control group. (D) Night‐time melatonin group showing dendrites of Purkinje cell as they extend into the molecular layer, nearly like that of control group. (E) Flow chart represent Mean ± SEM of dendrite length of Purkinje cell. All data were analyzed using One‐way ANOVA followed by Bonferroni post‐hoc test. a: statistically significant compared to control, b: statistically significant compared to MSG group, c: statistically significant compared to Day‐time melatonin group.

**Figure 8:**
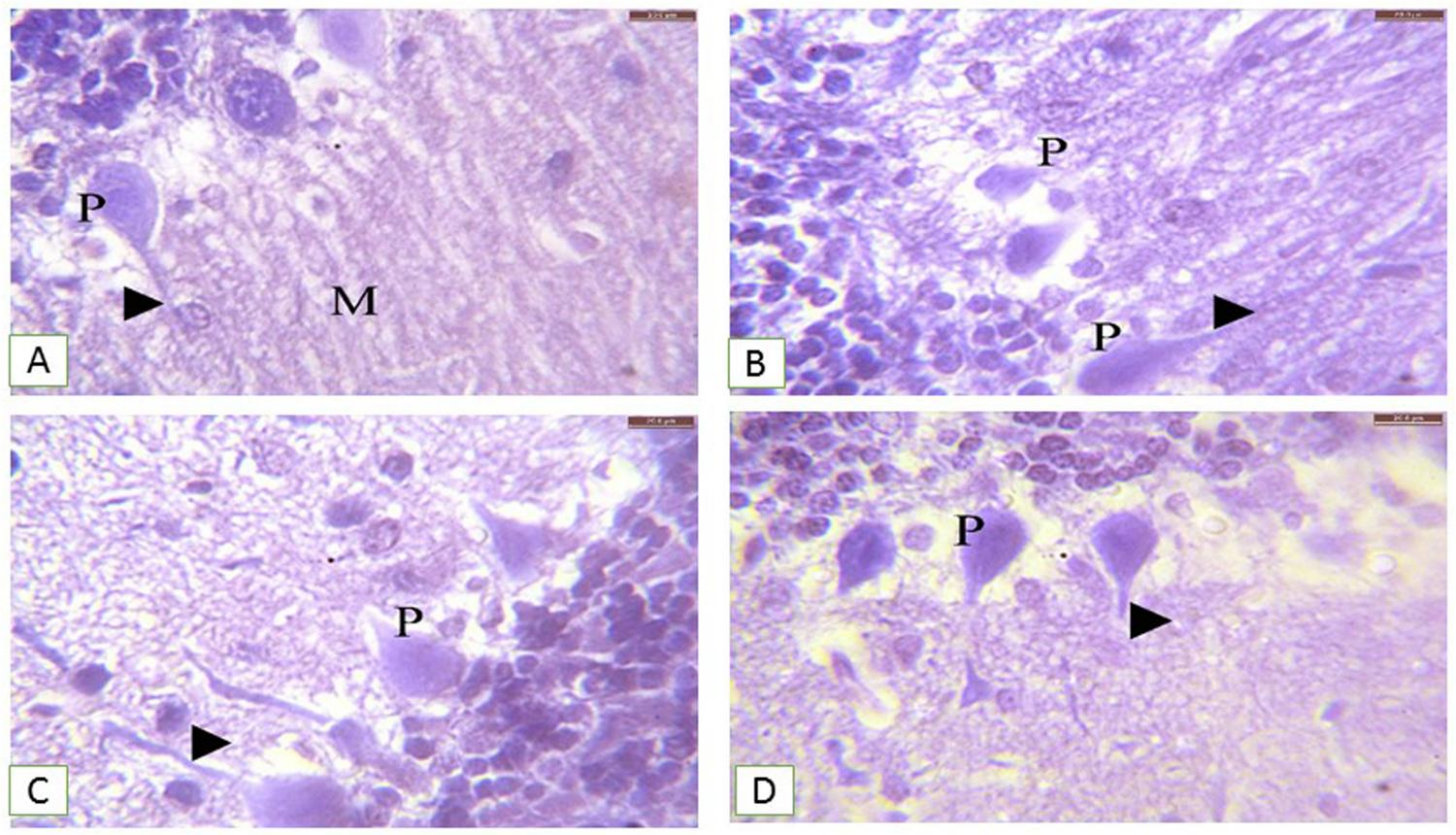
A photomicrograph of sections with the PTAH stain in the cerebellar cortex [PTAH stain X1000]. (A) A higher magnification showing the control group of the Purkinje cell layer (P), which is showing delicate arborizations (▶) up to molecular layer(M). (B) MSG group showing decreased length arborizations (▶) of the Purkinje cell(P). (C) Day time melatonin group shows restoration of the normal Purkinje cells (P) length arborizations (▶). (D) Night time melatonin group shows restoration of the normal Purkinje cells (P) length arborizations (▶), more than day group.

The cerebellar cortex of control group showed delicate dendritic arborizations of Purkinje cells extending up into molecular layer (Figures 7A,E and 8A). There were decreased in the Purkinje cell dendrites length by PTAH stain (Figures 7B,E and 8B). The cerebellum of the rats treated with melatonin by day showed dendritic length of Purkinje cells but less than the night‐time melatonin group (Figures 7C,E and 8C). The cerebellum of the rats treated with melatonin by night showed restoration of the normal architecture of the cortex like that of the control group and the dendrites of Purkinje cell, nearly like that of control group (Figures 7D,E and 8D).

#### 3.2.3 Immunohistochemistry Staining for Cyclo‐Oxygenase 2 (COX2) Protein

**Figure 9:**
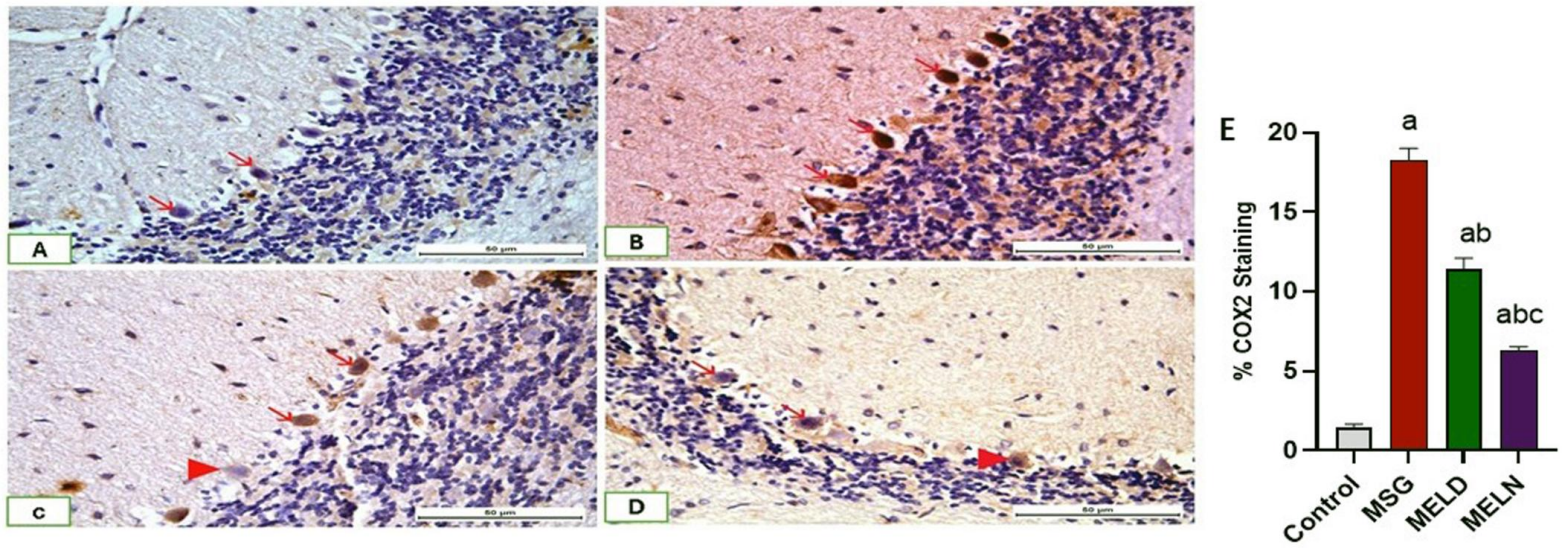
Photomicrographs of COX2 in the cerebellum [COX2 ×400]. (A) Control group showing negative immunoreaction, (B) MSG group showing increased positive brownish immunoreaction, (C) Day‐ time group showing shows negative immunoreaction in some of the Purkinje cells and another Purkinje cell shows positive reaction, (D) Night‐time group showing a negative immunoreaction in one Purkinje cell and some Purkinje cells show positive reaction. N.B: red arrow indicating the positive immunoreaction in some cells of the Purkinje cells and the (head arrow) indicating the negative immunoreaction. (E) Flow chart represent Mean ± SEM of the area percentage of COX2 immunostaining. All data were analyzed using One‐way ANOVA followed by Bonferroni post‐hoc test. a: statistically significant compared to control, b: statistically significant compared to MSG group, c: statistically significant compared to day‐time melatonin group.

Data showed a strong positive reaction of COX2 protein in MSG group as shown in (Figure 9B). The reaction of COX2 has increased positive brownish immunoreaction in some cells of the Purkinje cells when compared to control group (Figure 9A), which confirms a state of inflammation in the cerebellum by MSG, also has a significance when compared to both melatonin treated groups (*p* < 0.05) confirming the potential protective impact of melatonin against inflammation especially night‐time melatonin group as there is a strong significance between the morning and night‐time melatonin groups and the inflammatory reaction in the night‐time group (Figure 9D) is significantly less than the day‐time group (*p* < 0.05) (Figure 9C,E).

### 3.3 Normalization of Pro‐Inflammatory iNOS Protein Expression

**Figure 10:**
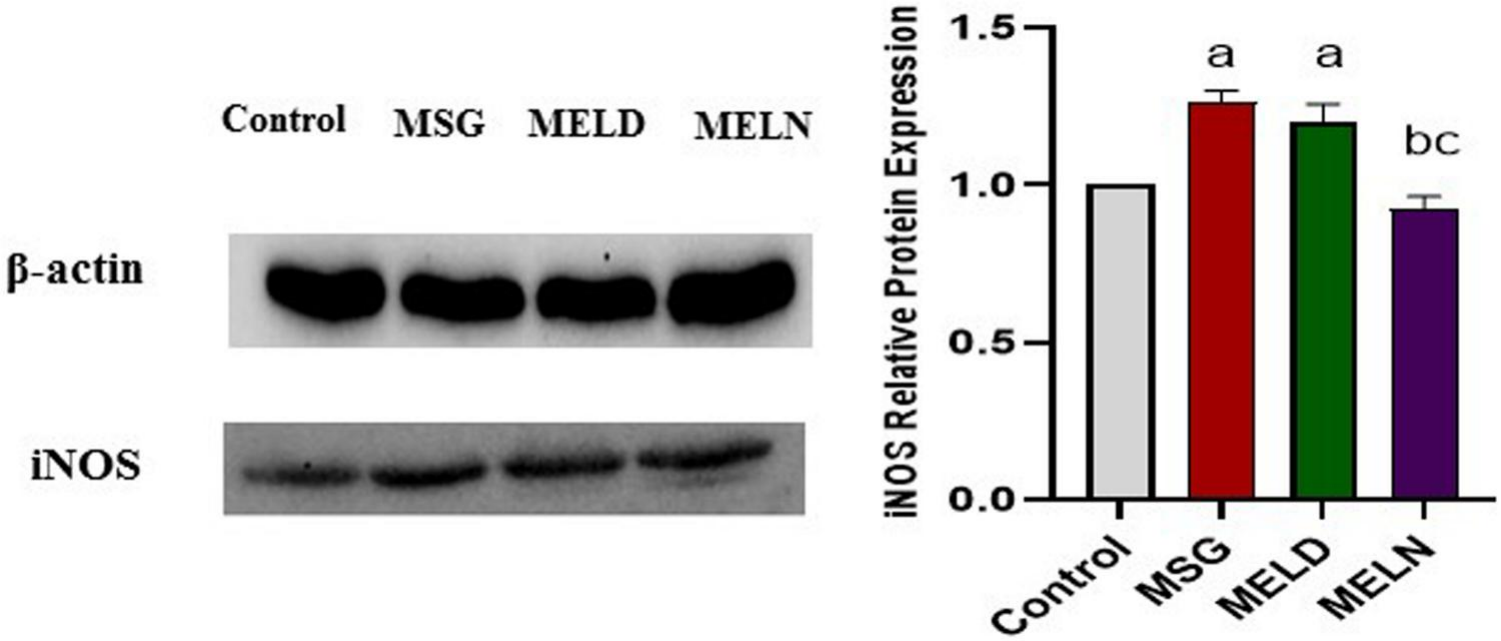
Modulation of cerebellar iNOS protein expression by melatonin in the MSG neurotoxicity model. (A) Quantitative analysis and (B) Representative Western blot images of iNOS protein levels. β‐actin was used as a loading control. Bars represent mean ± SEM. Data are presented as fold change relative to the control group. Statistical analysis: One‐way ANOVA followed by Bonferroni post‐hoc test. a: *p* < 0.05 versus control group; b: *p* < 0.05 versus MSG group; c: *p* < 0.05 versus day‐time melatonin group.

Western blot analysis revealed that the elevated nitrosative stress was associated with increased expression of inducible nitric oxide synthase (iNOS), a key mediator of inflammatory signaling. iNOS protein levels were significantly elevated in the MSG group compared to the control (*p* > 0.01) (Figure 10A,B).

Melatonin treatment potently attenuated this increase. Notably, night‐time administration not only significantly reduced iNOS levels (*p* > 0.001 vs. MSG) but also restored them to a level indistinguishable from the control group. A direct comparison between melatonin treatment times revealed a highly significant difference, with the night‐time group showing a substantially greater reduction in iNOS than the day‐time melatonin group (*p* > 0.001), underscoring the superior efficacy of night‐time melatonin administration in suppressing this pro‐inflammatory protein (Figure 10A,B).

### 3.4 Suppression of MSG‐Induced Pro‐Inflammatory Gene Upregulation

Administration of MSG resulted in a pronounced inflammatory response, as evidenced by the significant overexpression of key pro‐inflammatory mediators.

**Figure 11:**
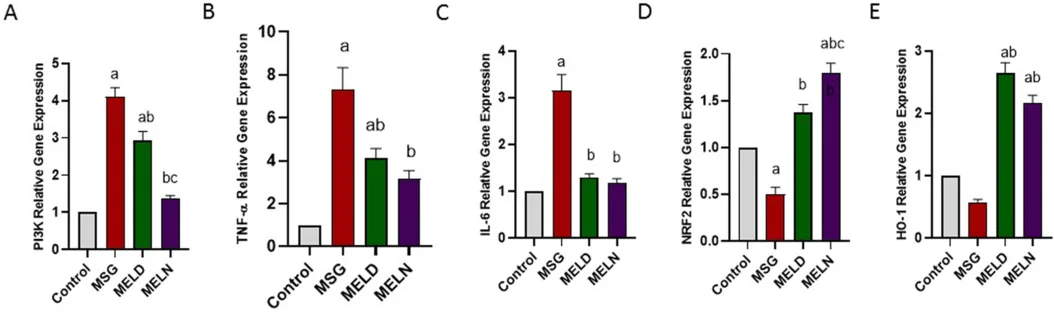
Modulation of cerebellar gene expression by melatonin in the MSG neurotoxicity model. qPCR analysis demonstrating the effects on (A) PI3K, (B) TNF‐α, (C) IL‐6, (D) HO‐1, and (E) NRF2 mRNA levels. Bars represent mean ± SEM. Data presented as fold change relative to the control group (*n* = 8 per group). Statistical analysis: One‐way ANOVA followed by Bonferroni post‐hoc test. a: *p* < 0.05 versus control group; b: *p* < 0.05 versus MSG group; c: *p* < 0.05 versus day‐time melatonin group.

PI3K: Expression was increased in the MSG group compared to the control (*p* < 0.001). Melatonin treatment significantly attenuated this increase. The day‐time treatment group showed a partial reduction (*p* < 0.05 vs. MSG), while the night‐time treatment group completely normalized PI3K expression to control levels (*p* = 1.00 vs. control). The therapeutic effect of night‐time melatonin was significantly greater than that of day administration (*p* < 0.05) (Figure 11A).

TNF‐α: A dramatic increase in TNF‐α expression was observed with MSG treatment (*p* < 0.001 vs. control). Both melatonin regimens significantly reduced expression (*p* < 0.05 vs. MSG). The level in the MELN group was not statistically different from the control, indicating near‐complete normalization (Figure 11B).

IL‐6: MSG induced a significant increase in IL‐6 expression (*p* < 0.01 vs. control). Melatonin treatment, regardless of timing, effectively downregulated IL‐6 to levels indistinguishable from the control (*p* < 0.01 vs. MSG) (Figure 11C).

### 3.5 Enhancement of Antioxidant Gene Expression and Pathway Activation

Concurrent with its anti‐inflammatory action, melatonin potently activated the endogenous antioxidant defense system, reversing the suppression caused by MSG.

NRF2: The expression of nuclear factor erythroid 2–related factor 2 (NRF2), the master regulator of the antioxidant response, was halved by MSG treatment (*p* < 0.05 vs. control). Melatonin significantly restored NRF2 levels (*p* < 0.01 vs. MSG). Notably, night‐time melatonin administration produced a significantly stronger upregulation than day‐time treatment (*p* < 0.05), elevating NRF2 expression above baseline control levels (Figure 11D).

HO‐1: The expression of the cytoprotective enzyme heme oxygenase‐1 (HO‐1) was significantly reduced by over 40% in the MSG group (*p* < 0.01 vs. control). Melatonin co‐treatment not only reversed this suppression but induced a robust upregulation, more than doubling HO‐1 expression compared to the control (*p* < 0.01 vs. both control and MSG) (Figure 11E).

### 3.6 Night‐time Administration Potentiates the Restorative Effects of Exogenous Melatonin on MSG‐Induced Suppression of Circadian Secretion in Rats

**Figure 12:**
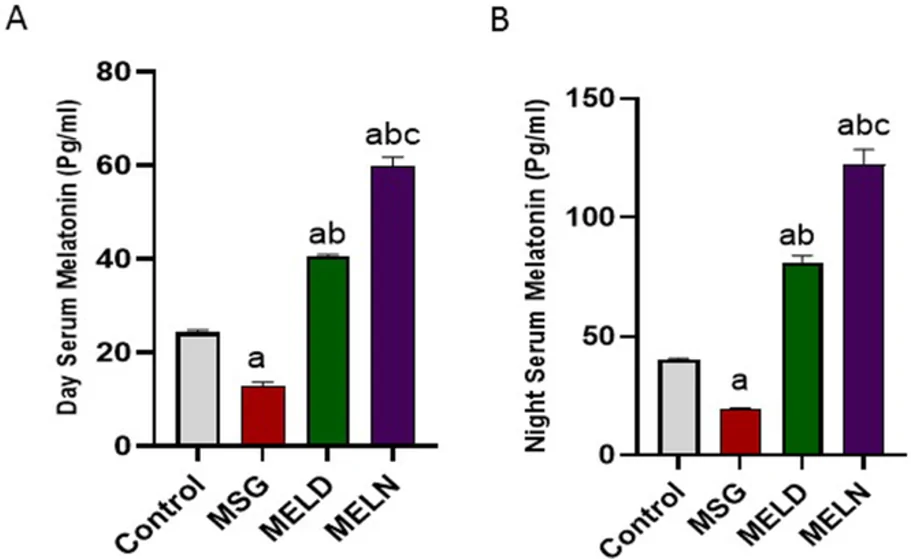
Circadian variations of serum melatonin across experimental groups. Data are presented as mean and SEM values for morning and night measurements in the Control, MSG, MELD, and MELN groups. Inter‐group comparisons were performed using One‐way ANOVA followed by Bonferroni post‐hoc test, while intra‐group differences between morning and night‐time points were assessed using paired *t*‐tests. a: *p* < 0.05 versus control group; b: *p* < 0.05 versus MSG group; c: *p* < 0.05 versus (MELD) group.

This study examined the circadian profile of plasma melatonin and the effects of monosodium glutamate (MSG) and exogenous melatonin administration in rats. MSG treatment caused a significant suppression of endogenous melatonin, reducing levels from 24.33 ± 0.5 pg/mL (day) and 39.9 ± 0.8 pg/mL (night) in the control group to 12.75 ± 0.9 pg/mL (day) and 19.4 ± 0.4 pg/mL (night) (*p* < 0.01) (Figure 12A,B).

Exogenous melatonin administration reversed this deficit and stimulated further secretion. The day‐time melatonin group achieved levels of 40.5 ± 0.4 pg/mL (day) and 80.8 ± 3.2 pg/mL (night). The night‐time treated group showed a more pronounced increase, reaching 59.7 ± 2.0 pg/mL (day) and 122.7 ± 5.8 pg/mL (night), significantly higher than the other groups (*p* < 0.01) (Figure 12A,B).

Consistent with its circadian rhythm, melatonin levels were significantly higher at night than during the day across all groups (*p* < 0.05). These results demonstrate that MSG disrupts melatonin production, which is effectively corrected and potentiated by exogenous supplementation. The superior efficacy of nighttime administration aligns with physiological secretion patterns and likely underlies its enhanced therapeutic antioxidant and anti‐inflammatory actions.

## 4 Discussion

The research demonstrated the role of administration timing of melatonin in cerebellar ataxic rat models and investigated its possible therapeutic potential in improving motor behavioral deficit and cerebellar pathology. Melatonin had a significant neuroprotective impact, as indicated by better motor performance, preservation of cerebellar architecture, attenuation of inflammatory indicators, restoration of antioxidant enzyme activities, downregulation of the pro‐inflammatory genes and activation of protective genes (iNOS and NRF2/HO‐1). Importantly, the therapeutic advantages of melatonin were more prominent at night than in the day‐time, indicating that circadian rhythm may play a role in improving its efficacy. The night‐time group showed near‐normalization of behavioral outcomes, Purkinje cell integrity, oxidative stress indicators, and endogenous melatonin secretion.

### 4.1 The role of melatonin on behavioral regulation

Melatonin had demonstrated a significant improvement and neuroprotective role in the all the deteriorated motor parameters in MSG group including motor behavior and coordination. The study showed that melatonin had improved the latency time and decreased the number of falls on the narrow beam in both treated melatonin groups especially the night‐time group which is consistent with Rehman et al. who studied the effect of melatonin in a mouse model of traumatic brain injury, melatonin treatment significantly improved complex motor coordination as assessed by a beam‐walking test when compared to the non‐treated group [24]. In addition to Gutierrez‐Valdez et al. who demonstrated that rats given chronic oral melatonin for 1 month began to exhibit a noticeable improvement of the alterations caused by the neurotoxin 6‐OHDA in beam traversal time around day 21 after treatment in Parkinson's disease [25]. Also Patki and Lau showed that melatonin dramatically improved motor coordination and balance on a narrow‐beam test in Parkinson's disease model [26].

The current study showed that night‐time melatonin administration had more significant improvement in motor performance in the open field test, which is consistent with Yang et al. which found that night‐day‐time melatonin injection rescued the depressive like behavior and improved the motor deficits in depression‐like behavior induced by constant light exposure in mice [27].

On assessing footprint test, our study revealed that melatonin improved the wide base width and the overlapping in both treated melatonin groups; especially the night‐time treated group, which is consistent with Jantzie et al. who reported that melatonin, either alone or in combination with erythropoietin, improved paw contact area in adult rats with preterm brain damage after a prenatal injury [28]. In addition to Rasheed et al. who showed normal paw contact/pressure, increased gait symmetry, and reduced ataxia—effects following melatonin administration in Parkinson's disease‐induced neurodegeneration model [29].

In accordance with Estrada‐Reyes et al., the effectiveness of melatonin is greatly influenced by when it is administered. Melatonin administered 1 h prior to the dark phase's onset was effective in antidepressant‐like behavioral experiments, indicating that dosing in accordance with endogenous melatonin onset improves efficacy when giving melatonin to the mice model by a dose of 4 mg/kg after either a single or a three‐dose protocol [30]. A previous study demonstrated night‐time melatonin administration was sufficient in the activation of MT_1_ signaling at night following constant light exposure in mice [31]. Another explanation was the synchronization of endogenous melatonin, emphasizing the superiority of administering melatonin at night‐time [32].

On the other hand, Yildirim et al. found that melatonin didn't improve significantly the motor deficit in the 6‐OHDA induced Parkinson's disease model and this could be attributed to the long duration of 6‐OHDA induction and the high level apoptosis of the dopaminergic neurons with the evidence that melatonin had no effect on NF‐κB which has a great role in limiting the transcription of the proinflammatory genes as IL‐6 and TNF‐α [33].

### 4.2 The Comparative Effect of Melatonin Administration (Day Versus Night) on Cerebellar Morphological Alterations

The motor deficits in the current study can be explained by the significant cerebellar neuronal damage and the oxidative stress pathway. In the current study, MSG induced significant cerebellar damage including a widely distorted and shrunken cerebellar Purkinje cell layer. The most obvious pathological alterations of this cerebellar injury were Purkinje cell necrosis, which was found in every animal in this group. The cerebellar pathological alteration was accompanied by decreased length of the Purkinje dendrites, these findings are in alignment with other studies [34, 35]. Additional oxidative and lysosomal stress further contributed to Nissl granules degeneration [36, 37, 38, 39, 40].

Our study found that melatonin had a significant restoration of the histological structure of the cerebellum in both melatonin treated groups; especially the night‐time treated group. The treated groups have fewer vacuoles in the Purkinje cell layer and better‐preserved lamination which is consistent with Essawy et al. who used melatonin in male rats with a dose of (10 mg/kg) daily for 4 weeks [10], and also with Abd El‐Hay et al. who used a dose of (10 mg/kg) daily and found that melatonin had a neuroprotective effect on the cerebellum by fewer shrunken pyknotic Purkinje cells, decreased vacuolization, and more typical astrocyte morphology [34]

Our study also found that melatonin had preserved the dendritic arborizations of Purkinje cells extending up into molecular layer in both melatonin treated groups with is also consistent with Cho et al. who used melatonin (20 mg/kg, i.p., 4 times before CA and 3 times after CA) [41] and also is consistent with Abd El‐Hay et al. showed that melatonin treatment resulted in intact Purkinje cell morphology and reduced degenerative ultrastructure; which revealed less axonal and neuropil degeneration [34].

One of the most significant alterations in the motor deficits was the increase of COX2, the inducible isoform of cyclooxygenase responsible for prostaglandin production which is consistent with several studies [42, 43]. This elevation in MSG group can be explained as glutamate excitotoxicity promotes COX2 via NMDA receptor‐mediated calcium influx, activating MAPK (ERK, JNK, p38) and NF‐κB pathways and promoting gene transcription [42, 43, 44]. Elevated COX2 promotes prostaglandin E2 (PGE2) production, which amps up neuroinflammation, improves glutamate release, and further destabilizes calcium homeostasis, causing a vicious cycle of excitotoxic damage [45, 46, 47].

Our study found a significant reduction of the area percentage of COX2 reaction was evaluated by densitometry scanning in both treated melatonin groups, mostly the night time melatonin group which is consistent with previous studies. Xie et al. proved that melatonin decreases COX‐2 expression and prostaglandin synthesis during spinal cord damage in the human studies, which helps to reduce apoptosis and enhance histology [48]. Shrestha et al found co‐treatment curcumin with melatonin reduced COX‐2 expression and tumor growth, demonstrating that melatonin can downregulate COX‐2 in tissue in tumor cells in a mice model with (10 mg/kg) for 11 days melatonin [49].

In the present study, melatonin can reduce COX2 levels at different level, melatonin and its metabolites operate as direct antioxidants, scavenging ROS/RNS and reducing lipid peroxidation and mitochondrial damage this results in melatonin suppresses MAPK phosphorylation and NF‐κB DNA binding, resulting in reduced COX‐2 transcription [50].

### 4.3 The Comparative Effect of Melatonin Administration (Day Versus Night) on Proinflammatory Markers Expression

A critical finding was the differential efficacy of melatonin in suppressing this pathway. While both treatment schedules normalized total NO levels, night‐time administration was strikingly more effective in reducing iNOS protein expression, bringing it down to control levels and showing a highly significant advantage over day‐time treatment. This can be attributed to chronopharmacology, melatonin's anti‐inflammatory actions, including the inhibition of NF‐κB and STAT3 signaling pathways that drive iNOS transcription, are likely gated by circadian rhythms [51]. Receptor sensitivity (MT1/MT2) and downstream signaling cascades are maximal during the biological night, allowing for more potent suppression of inflammatory gene expression [52]. The normalization of total NO despite a more potent iNOS knockdown in the MELN group may reflect contributions from constitutive NOS isoforms or the scavenging of reactive nitrogen species, which are not fully captured by stable end‐product assays [53, 54].

The gene expression data reveal a significance pattern of melatonin's regulatory influence. MSG triggered a robust pro‐inflammatory response, with a dramatic 4‐fold increase in PI3K and a 7.3‐fold increase in TNF‐α. The complete normalization of PI3K expression only in the MELN group is a standout result. This aligns with the concept that the crosstalk between circadian clock components (e.g., BMAL1) and signaling pathways like PI3K is time‐sensitive [55]. Night‐time melatonin, by optimally synchronizing circadian rhythms, may more effectively decouple the aberrant PI3K/Akt/NF‐κB activation that exacerbates inflammation under excitotoxic stress [56].

Both TNF‐α and IL‐6 were significantly downregulated by melatonin, with night‐time administration showing a trend toward greater efficacy for TNF‐α. This broad anti‐cytokine effect is consistent with melatonin's inhibition of the master regulator NF‐κB [57]. Conversely, the potent upregulation of antioxidant genes NRF2/HO‐1 highlights melatonin's proactive role in boosting endogenous defenses. The significant, supra‐normal induction of HO‐1 by both treatments underscores a powerful cytoprotective response, generating antioxidant bilirubin and anti‐inflammatory carbon monoxide [58]. The significantly stronger upregulation of the master regulator NRF2 in the night treated group is pivotal. It suggests that the activation of the NRF2/HO‐1 axis, a cornerstone of cellular defense, is subject to circadian optimization, maximizing the transcriptional activation of a battery of antioxidant and detoxifying genes during the night [59, 60].

### 4.4 The Comparative Effect of Melatonin Administration (Day Versus Night) on Serum Hormone Level

The serum melatonin results provide the foundational explanation for the observed time‐of‐day effects. MSG significantly suppressed both day‐time and nocturnal melatonin secretion, attenuating but not eliminating the rhythm. This likely results from excitotoxic damage to the suprachiasmatic nucleus (SCN), the central circadian pacemaker that drives pineal secretion, and/or direct inhibition of pinealocyte function [61].

Exogenous melatonin not only corrected this deficit but produced a pharmacologically potent, rhythm‐aligned increase. The night‐time administration generated a significantly higher amplitude rhythm than both the control and day‐time melatonin groups. This demonstrates that delivering melatonin in phase with the endogenous secretory peak leads to higher bioavailability and optimal engagement of its receptors, which themselves exhibit circadian variation in expression and sensitivity [62, 63]. This restored and amplified circadian signal is the likely driver of the superior therapeutic outcomes—enhanced receptor‐mediated activation of pathways like SIRT1, more effective suppression of NF‐κB, and stronger induction of NRF2 [52].

## 5 Limitation

The study evaluated the therapeutic potential of melatonin with different administration time in adult male rats so further studies are required to investigate its influence on female rats. Long‐term studies are also needed to evaluate if night‐time melatonin administration could be a preventive measure against continuous MSG intake. Furthermore, future studies could explore the in vitro effects of MSG and night‐time melatonin administration in more detail to complement the in vivo findings and further elucidate the underlying cellular mechanisms including receptor level analysis.

## 6 Conclusion

**Figure 13:**
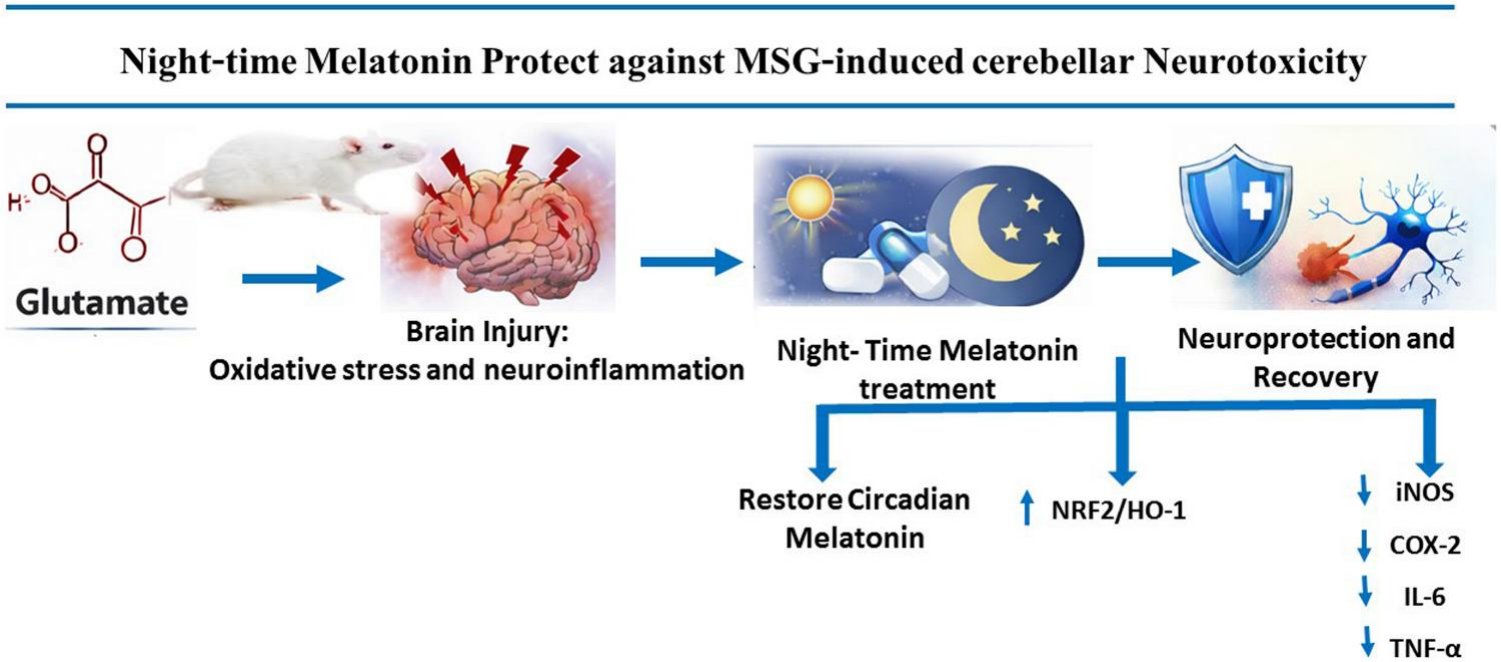
Summarizes the mechanisms behind modulation of neurobehavioral dysfunctions, cerebellar abnormalities of night‐time melatonin on MSG‐induced cerebellar ataxia. Exogenous melatonin, especially when given at night, provides strong neuroprotective benefits by influencing circadian serum melatonin levels. It inhibits PI3K‐dependent inflammatory signaling and encourages the nuclear translocation of NRF2, along with activation of the NRF2/HO‐1 antioxidant pathway. Consequently, reduces inflammatory mediators (↓ iNOS, ↓ COX‐2, ↓IL‐6, and ↓INF‐α). The figure was designed by PowerPoint program with the use of figure sources from chat GPT.

In summary, the results demonstrate that MSG‐induced cerebellar neurotoxicity is mediated through a concerted attack on redox balance and inflammatory signaling. Melatonin acts as a multifaceted countermeasure, scavenging oxidants, quenching inflammation, and potently upregulating endogenous antioxidant genes. Crucially, the efficacy of this intervention is chronodependent. Administration during the night, coinciding with the peak of endogenous circadian physiology, optimizes melatonin's bioavailability and receptor‐mediated actions, leading to significantly greater suppression of key inflammatory mediators like iNOS and PI3K, and a more robust activation of the master antioxidant regulator NRF2. This study strongly advocates for the consideration of chronotherapeutic strategies, utilizing night‐time melatonin supplementation, to maximize neuroprotection in excitotoxic and inflammatory disorders. (Figure 13)

## Author Contributions

**Alaa Gamal Ahmad:** methodology, investigation, writing – original draft, software. **Noha M. Abd El‐Fadeal:** conceptualization, supervision, writing – review and editing, software, formal analysis. **Horeya Erfan Korayem Arafat:** software, supervision, writing – review and editing, visualization, formal analysis, conceptualization. **Dalia Ibrahim Ahmed:** supervision, conceptualization, writing – review and editing, visualization, formal analysis. **Rasha Atta:** supervision, conceptualization, writing – review and editing, software, formal analysis.

## Ethics Statement

Ethical approval for this study was obtained from the “Research Ethics Committee of the Faculty of Medicine,” Suez Canal University, Egypt on 24\02\2024 (approval code 5595#). All animal procedures were conducted in accordance with the “National Institutes of Health Guide” for the Care and Use of Laboratory Animals (NIH Publications No. 8023, revised 1978).

## Data Availability

The data that support the finding of the study will be available on request from the corresponding author.
